# Decoding Immune Dysregulation in Childhood Idiopathic Nephrotic Syndrome: Insights From Lymphocyte Subsets and Urinary Cytokine Analysis

**DOI:** 10.1155/ijne/3601660

**Published:** 2026-11-04

**Authors:** A. K. Anju Ashok, Gautom Kumar Saharia, Amit Kumar Satapathy, Manaswini Mangaraj

**Affiliations:** ^1^ Department of Biochemistry AIIMS Bhubaneswar 751019 Odisha India aiims.edu; ^2^ Department of Pediatrics AIIMS Bhubaneswar 751019 Odisha India aiims.edu

**Keywords:** B cell, cytokines, immunopathogeneisis, lymphocyte, nephrotic syndrome, T cell

## Abstract

**Background:**

Idiopathic nephrotic syndrome (INS) is a primary kidney disease of childhood involving podocytes where immune dysregulation is thought to play a major role in pathogenesis. The aim of the study was to evaluate peripheral lymphocyte subsets and urinary cytokines in children with INS to understand the immune mechanisms and their association with steroid responsiveness.

**Methods:**

This was a prospective longitudinal observational study of children with INS, conducted at a tertiary care hospital from August 2021 to June 2023. A total of 34 children with INS (17 steroid sensitive [SSNS] and 17 steroid resistant [SRNS]) and 17 healthy controls were enrolled. Flow cytometry analyzed CD3+, CD4+, CD8+, and CD19+ lymphocyte subsets, while ELISA estimated urinary IFN‐γ, IL‐4, and IL‐13 at the onset of disease/relapse and during remission.

**Results:**

Children with INS showed significantly higher counts of CD3+, CD4+, CD8+, and CD19+ cells compared to controls (*p* < 0.05) at the time of disease onset. SRNS patients had notably higher counts of all these subsets than SSNS patients. Following steroid therapy, CD19+ and CD3+ counts increased in both groups; however, SRNS patients also showed a persistent increase in CD4+ cells and urinary IFN‐γ and IL‐13, suggesting ongoing immune activation. ROC curve analysis identified CD3+, CD4+, and CD19+ counts with moderate diagnostic performance with AUC values ranging from 0.706 to 0.747.

**Conclusion:**

Significant alterations in T‐ and B‐lymphocyte subset populations indicate immune activation in the pathogenesis of childhood INS. The distinct immunological profiles and poststeroid cytokine increases in SRNS patients indicate persistent local immune activation despite treatment.

## 1. Introduction

Idiopathic nephrotic syndrome (INS) represents the majority of nephrotic syndrome cases in children. Its annual incidence is estimated at 9–16 per 100,000 children in Asia, compared with 2–7 per 100,000 worldwide [1]. Boys are more frequently affected than girls, with a male‐to‐female ratio of approximately 3:1 [2]. The epidemiological pattern in Indian children parallels that observed in cohorts from the United States of America and Europe. Histologically, minimal change disease accounts for about 80% of the cases, with boys comprising nearly 70% of those affected. Most children with INS present between 2 and 6 years of age [3, 4]. Corticosteroids remain the cornerstone of therapy, and classification is based on steroid responsiveness into steroid‐sensitive nephrotic syndrome (SSNS) and steroid‐resistant nephrotic syndrome (SRNS) [5].

The precise pathogenesis of INS remains elusive. Being a primary glomerular disease, it can be best defined as podocytopathy, which involves a loss of podocytes, leading to complete effacement of foot process. However, systemic factors (i.e., immune‐mediated or circulating mediators) are thought to be the major contributors to this podocyte effacement [6]. Dysregulation or dysfunction of T lymphocytes is assumed to play a role in its genesis [7]. Alteration of B lymphocytes, natural killer (NK) cells, and NK T cells has also been registered in a few studies. In addition, released cytokines from T cells filtered through the glomerulus damage the podocytes, affecting the glomerular filtration barrier. Even though T helper 1 (Th1) cytokines are associated with the disease, T helper 2 (Th2) cytokines were also found to increase significantly, where Interleukin‐4 (IL‐4) and Interleukin‐13 (IL‐13) behave as circulatory permeability factors [8].

Although abnormalities in T‐ and B‐cell populations and cytokine signaling have been reported in childhood INS, the relationship between peripheral immune‐cell profiles and steroid responsiveness remains incompletely understood. Furthermore, relatively few studies have simultaneously evaluated circulating lymphocyte subsets and urinary cytokines in children with SSNS and SRNS, particularly using paired assessment during active disease and remission. Characterizing these immunological differences may provide insights into the biological heterogeneity underlying steroid responsiveness. This study intends to understand the alteration of lymphocyte subpopulation in peripheral blood along with an estimation of urinary cytokines like Interferon (IFN)‐γ and IL‐4 and IL‐13 in cases of both SSNS and SRNS compared to controls, to know the immune mechanism behind the disease. Primary objective of the study is to detect the alteration of blood lymphocyte subpopulation in INS cases with CD3, CD45, CD4, CD8, and CD19 assay in comparison to healthy controls using flow cytometry and estimate the circulating cytokines (IFN‐γ, IL‐4, and IL‐13) in relation to proteinuria.

## 2. Methodology

### 2.1. Study Design

This was a prospective longitudinal observational study of children with INS, with a healthy control group included for comparison of immunological parameters. Children with INS were enrolled at disease onset or relapse before the initiation of corticosteroid therapy and were subsequently classified as SSNS or SRNS according to their response to standard corticosteroid therapy. The study was conducted at a tertiary care hospital from August 2021 to June 2023. Children aged 1–14 years diagnosed with INS according to KDIGO criteria were enrolled in the study following parental consent and, where appropriate, child assent [9]. However, children with secondary nephrotic syndrome (e.g., lupus nephritis and infections), congenital nephrotic syndrome, seriously ill and organ failure, sepsis, and positive family history were excluded. Enrolled children were categorized into three groups. Group A (SSNS): patients with INS who achieved remission, defined as negative or trace proteinuria on urine dipstick for three consecutive days after 4 weeks of standard steroid therapy. Group B (SRNS): patients with INS who failed to achieve remission despite 4 weeks of standard steroid therapy. Group C (Controls): apparently healthy children attending the pediatric outpatient department for growth monitoring or immunization. Children with any comorbidities were excluded from the control group. For controls, blood and urine samples were collected prior to the administration of immunization.

### 2.2. Ethical Clearance

Ethical clearance has been obtained from the Institutional Ethics Committee (IEC) of AIIMS, Bhubaneswar with Approval Ref. no. IEC/AIIMS BBSR/PG Thesis/2021‐22/60 dated 13 Aug 2021.

### 2.3. Study Procedure

Children diagnosed with INS attending the pediatric outpatient department were recruited. Diagnosis was established according to the Kidney Disease: Improving Global Outcomes (KDIGO) criteria [10]. All participants received standard prednisolone therapy at a dose of 2 mg/kg/day for 4 weeks. Children achieving remission, defined as per the criteria outlined above, were classified as SSNS, whereas those failing to achieve remission were categorized as SRNS.

After enrollment, detailed information on disease onset, course, prior treatments, drug history, and demographic characteristics was collected. Anthropometric measurements were recorded, and baseline laboratory investigations included complete blood count, liver function tests, and kidney function tests. Lymphocyte subset analysis and urinary cytokine evaluation were performed at two time points: (i) at the onset of the first episode prior to initiation of steroids, or at the onset of relapse before steroids in previously treated patients; and (ii) during remission, defined according to KDIGO criteria (urine protein–creatinine ratio < 0.2), following 4 weeks of prednisolone in SSNS or at corresponding time points in SRNS irrespective of PROTE (complete or partial remission per KDIGO) [9].For immunophenotyping, 2 mL of venous blood was collected in EDTA vacutainer tubes and processed within 6 hours of collection. Lymphocyte subsets were quantified using a ten‐color Navios flow cytometer (Beckman Coulter, Ireland, Inc., Lismeehan). Data acquisition was performed on the connected computer system and analyzed using the inbuilt CXP software (Figure 1). The following procedure of fluorescent tagged antibodies manufactured by Beckman Coulter Inc., USA was used in the study:

• Anti‐CD45 antibody tagged with Fluorescein isothiocyanate (FITC): CD45 is a transmembrane protein with tyrosine phosphatase activity, expressed by all lymphocytes.
• Anti‐CD3 antibody tagged with Phycocyanin 5 (PC5): T‐cell antigen receptor (TCR) association is accomplished by CD3, an essential membrane glycoprotein that is vital to TCR cell surface expression. So, CD3 is found in all mature T cells.
• Anti‐CD4 antibody tagged with Phycoerythrin (RD1): Helper T cells comprise CD4, a membrane glycoprotein that serves as a coreceptor by binding to major histocompatibility complex (MHC) molecules that the TCR identifies.
• Anti‐CD8 antibody tagged with electron coupled dye (ECD): CD8 is a membrane glycoprotein expressed on cytotoxic T lymphocytes, which also acts as a coreceptor.
• Anti‐CD19 Antibody tagged with Phycocyanin 7 (PC7): CD19 is a marker for B lymphocytes. They function as dominant signaling molecule among a multimolecular complex.
• To get the absolute count of each lymphocyte subset studied, flow counting beads were used.

**FIGURE 1:**
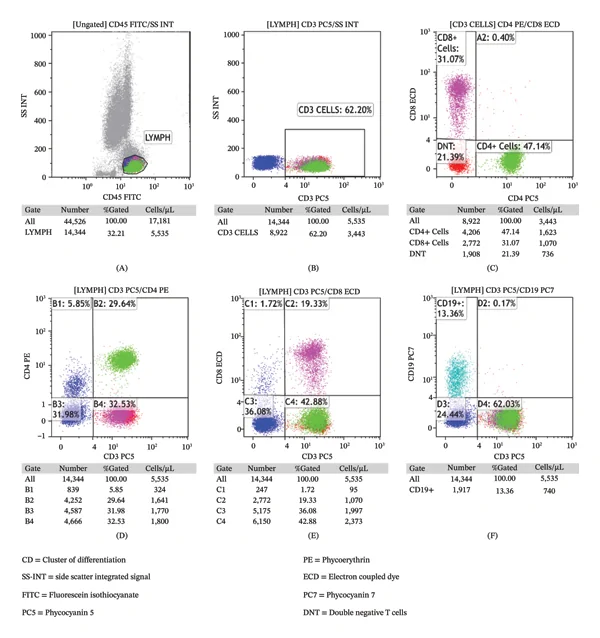
Lymphocyte subset analysis by flow cytometry with the gating strategy depicted.

Lymphocytes were gated based on forward and side scatter, followed by CD45+ selection. Subsequent gates identified CD3+, CD4+, CD8+, and CD19+ subsets.

Freshly voided urine samples of approximately 10 mL were collected from all participants. The collected urine was centrifuged at 3000 rpm for 20 min and supernatant without the sediment was used as per the instructions in the Enzyme‐Linked Immunosorbent Assay (ELISA) kit insert. A total of 500 μL of supernatant urine was stored at −80°C in labeled plastic microcentrifuge tubes, which was used for human interferon gamma (IFN‐γ), IL‐4, and IL‐13 level estimation by BioAssay Technology Laboratory (BT Lab, China) ELISA Kit. Precoated ELISA plates were used for respective parameters. All reagents, standard solutions, and samples were prepared as per manufacturer’s instructions. All the reagents were brought to room temperature before use. The assays were performed at room temperature and absorbance measured at 450 nm.

### 2.4. Statistical Analysis

Normality of continuous variables was assessed using the Shapiro–Wilk test. Normally distributed continuous variables were expressed as mean ± standard deviation and compared using Student’s *t*‐test. Chi‐square test was applied to assess categorical variables. A paired *t*‐test was employed to examine the mean difference between the pre‐ and post‐therapy samples. Using Pearson’s correlation analysis, the correlation between lymphocyte subsets, urine interleukin status, and disease progression was examined. Receiver operating characteristic (ROC) curve analysis was utilized to evaluate the diagnostic efficacy of the lymphocyte subpopulation and urine cytokines. *p* values less than 0.05 were regarded as significant. With SPSS 26.0, statistical analysis was carried out.

## 3. Results

A total of 34 children with INS were enrolled, comprising 17 with SSNS and 17 with SRNS, along with an equal number of healthy controls. The demographic profile of the cohort was consistent with Asian populations, with boys accounting for 70% (*n* = 24) and girls 30% (*n* = 10). The mean age of cases was 5.29 ± 3.26 years, while the control group included 17 children (13 boys) with a mean age of 5.65 ± 2.26 years. At baseline, children with INS had biochemical features consistent with active nephrotic syndrome, including hypoalbuminaemia, hypercholesterolemia, and significant proteinuria. These parameters were recorded to document the clinical state at the time of immunological sampling rather than as comparative outcome measures (Table 1). At baseline, total leukocyte counts were markedly higher in cases compared with controls. The mean leukocyte count was 18,804 ± 6410 cells/mm^3^ in SSNS, 11,431 ± 3606 cells/mm^3^ in SRNS, and 5505 ± 1232 cells/mm^3^ in the control group.

**TABLE 1:** Baseline demographic and clinical characteristics.

Parameter	Controls (*n* = 17)	Nephrotic syndrome (*n* = 34)	*p* value
Age (years), mean ± SD	5.65 ± 2.26	5.29 ± 3.26	0.72
Male sex, *n* (%)	13 (76%)	24 (71%)	0.74
Serum albumin (g/dL)	3.44 ± 0.27	1.71 ± 0.38	< 0.001
Total cholesterol (mg/dL)	124 ± 8	393 ± 63	< 0.001
Leukocyte count (cells/mm^3^)	5505 ± 1232	15,118 ± 5807	< 0.001
Serum creatinine (mg/dL)	0.4 (0.38–0.5	0.44 (0.36–0.48)	0.5
eGFR (ml/min/1.73 m^2^)	108 ± 22	100 ± 18	0.19

*Note:* eGFR: estimated glomerular filtration rate as calculated by using Schwartz formula.

### 3.1. Lymphocyte Subset

At the initial evaluation, children with SSNS demonstrated higher CD3+ counts (2660 ± 711 vs. 2135 ± 827 cells/mm^3^, *p* = 0.071) and CD4+ counts (1585 ± 523 vs. 1255 ± 395 cells/mm^3^, *p* = 0.058) compared with controls, though these differences did not reach statistical significance. CD8+ lymphocytes were significantly elevated in SSNS (1262 ± 377 vs. 881 ± 294 cells/mm^3^, *p* = 0.003), whereas CD19+ counts were comparable between groups (677 ± 291 vs. 631 ± 304 cells/mm^3^, *p* = 0.67). Urinary cytokine levels (IFN‐γ, IL‐4, and IL‐13) showed no significant differences between SSNS and controls (Table 2).

**TABLE 2:** Comparison of lymphocyte subsets and urinary cytokines between controls and SSNS.

Parameter	Controls (*n* = 17) mean ± SD	SSNS (*n* = 17) mean ± SD	*p* value
CD3+ cells (cells/mm^3^)	2135 ± 827	2660 ± 711	0.071
CD4+ cells (cells/mm^3^)	1255 ± 395	1585 ± 523	0.058
CD8+ cells (cells/mm^3^)	881 ± 294	1262 ± 377	0.003
CD19+ cells (cells/mm^3^)	631 ± 304	677 ± 291	0.67
Urine IFN‐γ (ng/mL)	25.5 ± 30.4	39.4 ± 40.9	0.29
Urine IL‐4 (ng/L)	228 ± 80	261 ± 77	0.24
Urine IL‐13 (ng/L)	22.2 ± 6.5	21.0 ± 6.5	0.61

Abbreviation: SSNS, steroid‐sensitive nephrotic syndrome.

In contrast, children with SRNS exhibited significant elevations across all lymphocyte subsets compared with controls. CD3+ counts were markedly higher (3606 ± 1120 vs. 2135 ± 827 cells/mm^3^, *p* < 0.001) as were CD4+ (2108 ± 772 vs. 1255 ± 395 cells/mm^3^, *p* < 0.001), CD8+ (1635 ± 550 vs. 881 ± 294 cells/mm^3^, *p* < 0.001), and CD19+ cells (942 ± 405 vs. 631 ± 304 cells/mm^3^, *p* = 0.018). Urinary cytokine levels did not differ significantly between groups: IFN‐γ was comparable (28.9 ± 24.6 vs. 25.5 ± 30.4 ng/mL, *p* = 0.74), IL‐4 showed a nonsignificant trend toward elevation (279 ± 78 vs. 228 ± 80 ng/L, *p* = 0.09), and IL‐13 tended to be lower in SRNS (17.8 ± 5.2 vs. 22.2 ± 6.5 ng/L, *p* = 0.07) (Table 3).

**TABLE 3:** Comparison of lymphocyte subsets and urinary cytokines between control and SRNS.

Parameter	Controls (*n* = 17) mean ± SD	SRNS (*n* = 17) mean ± SD	*p* value
CD3+ cells (cells/mm^3^)	2135 ± 827	3606 ± 1120	< 0.001
CD4+ cells (cells/mm^3^)	1255 ± 395	2108 ± 772	< 0.001
CD8+ cells (cells/mm^3^)	881 ± 294	1635 ± 550	< 0.001
CD19+ cells (cells/mm^3^)	631 ± 304	942 ± 405	0.018
Urine IFN‐γ (ng/mL)	25.5 ± 30.4	28.9 ± 24.6	0.74
Urine IL‐4 (ng/L)	228 ± 80	279 ± 78	0.09
Urine IL‐13 (ng/L)	22.2 ± 6.5	17.8 ± 5.2	0.07

Abbreviation: SRNS, steroid‐resistant nephrotic syndrome.

SRNS patients exhibited notably elevated counts of CD3+, CD4+, CD8+, and CD19+ cells than SSNS (Table 4). Following four weeks of steroid medication, both case groups’ CD19+ and CD3+ cell counts dramatically increased. Whereas the SRNS group had only significant increase in CD4+ cells (*p* = 0.034), the SSNS group showed an increase in CD8+ cells (*p* = 0.028) (Table 5).

**TABLE 4:** Comparison between steroid‐sensitive and steroid‐resistant nephrotic syndrome.

Parameter	SSNS (*n* = 17)	SRNS (*n* = 17)	*p* value
Creatinine (mg/dL)	0.5 (0.4–0.6)	0.4 (0.34–0.5)	0.06
eGFR (ml/min/1.73 m^2^)	95 ± 17	104 ± 14	0.17
CD3+ cells (cells/mm^3^)	2660 ± 711	3606 ± 1120	< 0.001
CD4+ cells (cells/mm^3^)	1585 ± 523	2108 ± 772	< 0.001
CD8+ cells (cells/mm^3^)	1262 ± 377	1635 ± 550	0.030
CD19+ cells (cells/mm^3^)	677 ± 291	942 ± 405	0.027
Urine IFN‐γ (ng/mL)	39.4 ± 40.9	28.9 ± 24.6	0.44
Urine IL‐4 (ng/L)	261 ± 77	279 ± 78	0.42
Urine IL‐13 (ng/L)	21.0 ± 6.5	17.8 ± 5.2	0.10

Abbreviations: SRNS, steroid‐resistant nephrotic syndrome; SSNS, steroid‐sensitive nephrotic syndrome.

**TABLE 5:** Comparison of lymphocyte subset and cytokines pre and post steroid therapy.

Parameter	SSNS (*n* = 17)			SRNS (*n* = 17)		
	Mean difference (first visit–second visit)	Std. dev. of mean difference	*p* value (paired samples’ *t*‐test)	Mean difference (first visit–second visit)	Std. dev. of mean difference	*p* value (paired samples’ *t*‐test)
CD19 count (cells/mm^3^)	−1401.1	2246.3	**0.020**	−830.37	1385.37	**0.025**
CD3 count (cells/mm^3^)	−3518.5	5275.0	**0.014**	−3428.35	4862.33	**0.009**
CD4 count (cells/mm^3^)	−1744.8	3443.6	0.053	−1525.14	2713.07	**0.034**
CD8 count (cells/mm^3^)	−1132.8	1938.5	**0.028**	−1051.44	2089.36	0.054
Urine Interferon Gamma (pg/mL)	−16.5	52.8	0.216	−24.43	39.71	**0.022**
Urine Interleukin‐4 (pg/mL)	22.8	121.2	0.449	45.74	112.29	0.112
Urine Interleukin‐13 (pg/mL)	−1.2	9.0	0.593	−3.56	6.79	**0.046**

*Note:* The bold values represent statistically significant *p* value.

Abbreviations: SRNS, steroid‐resistant nephrotic syndrome; SSNS, steroid‐sensitive nephrotic syndrome.

### 3.2. Pearson’s Correlation Analysis

All the lymphocyte subsets showed a very strong positive correlation with each other in both the first and second patient visits. On the first visit, there was a high positive association amongst urinary cytokines and on the second visit, and there was a moderate positive correlation. CD3+ and CD19+ counts showed a moderate correlation, and CD8+ count showed a minor correlation with urinary IL‐4 on the first visit. None of the other urinary cytokines correlated with the lymphocyte subsets on both visits. The correlation heatmap (Pearson’s r) for immunological parameters is depicted in Figure 2.

**FIGURE 2:**
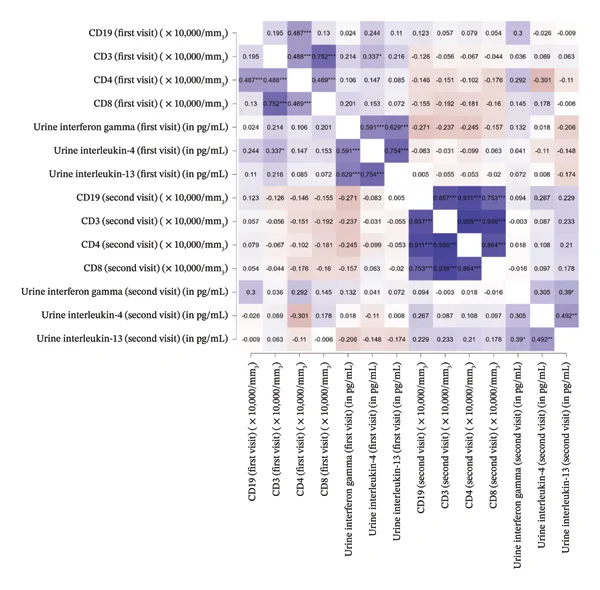
Correlation heatmap (Pearson’s *r*) for immunological parameters adjusted for groups.

### 3.3. ROC Analysis

At the initial visit, CD3+, CD4+, and CD8+ cells have a strong diagnostic efficacy for INS (*p* < 0.001). With an area under the curve of 0.865, CD8+ cells demonstrated the highest diagnostic efficiency of the three. At the patient’s initial visit, CD3+, CD4+, and CD19+ counts can be utilized to predict the likelihood that the patient could go on to develop steroid resistance (*p* = 0.006, 0.017,  and 0.023, respectively) (Table 6 and Figures 3 and 4).

**TABLE 6:** Receiver operating characteristic (ROC) curve analysis for diagnosing cases of nephrotic syndrome from healthy population and steroid‐sensitive from steroid‐resistant cases.

Parameters	For diagnosing control vs cases		For diagnosing steroid sensitive vs steroid resistant	
	Area	*p* value	Area	*p* value
CD19 (× 10,000/mm^3^)	0.635	0.106	0.706	**0.023**
CD3 (× 10,000/mm^3^)	0.773	**< 0.001**	0.747	**0.006**
CD4 (× 10,000/mm^3^)	0.784	**< 0.001**	0.716	**0.017**
CD8 (× 10,000/mm^3^)	0.865	**< 0.001**	0.657	0.103
Urine Interferon Gamma (pg/mL)	0.590	0.320	0.467	0.751
Urine Interleukin‐4 (pg/mL)	0.638	0.094	0.571	0.491
Urine Interleukin‐13 (pg/mL)	0.388	0.212	0.349	0.127

*Note:* The bold values represent statistically significant *p* value.

**FIGURE 3:**
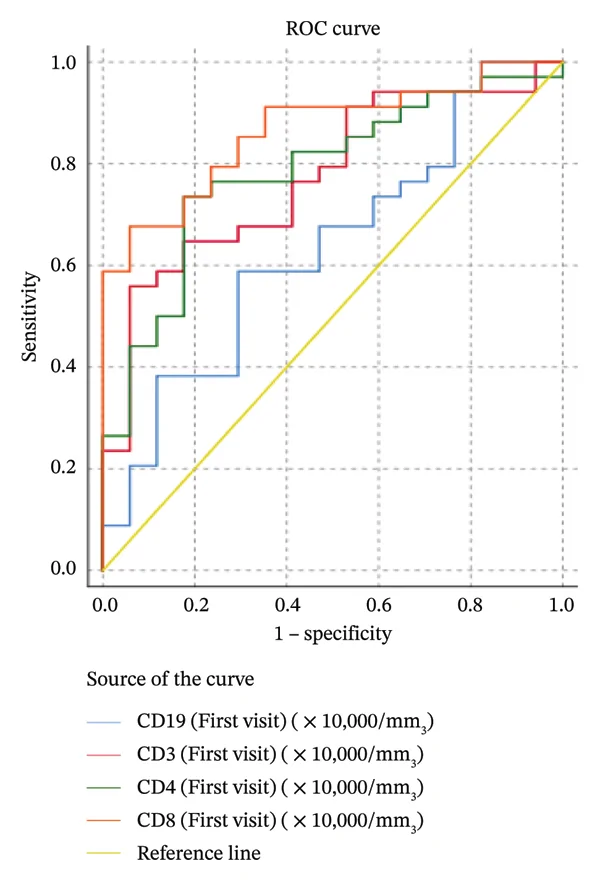
Receiver operating characteristic (ROC) curve analysis for lymphocyte subsets to diagnose cases of nephrotic syndrome from healthy controls.

**FIGURE 4:**
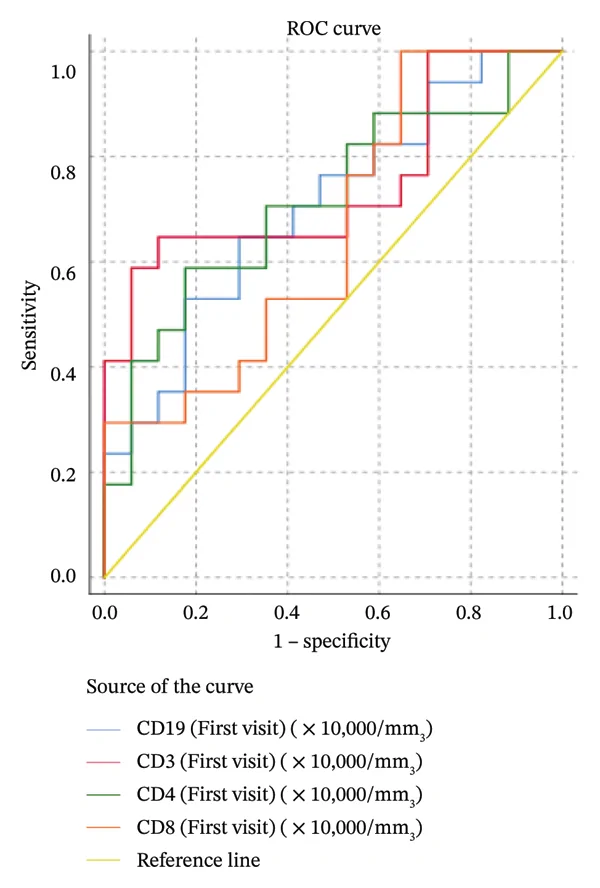
Receiver operating characteristic (ROC) curve analysis for lymphocyte subsets to diagnose cases of steroid resistant versus steroid sensitive nephrotic syndrome.

## 4. Discussion

INS in children is considered to be an immune dysregulation involving both cellular and humoral immunity resulting in podocyte injury and increased glomerular permeability. We evaluated alterations in peripheral blood lymphocyte subsets and urinary cytokines in children with INS at time of relapse and remission. We also explored the lymphocyte subset changes among children with SSNS and SRNS. Our findings suggest that INS is associated with measurable changes in immune cell populations, particularly T‐ and B‐lymphocyte subsets, which may reflect the immunological mechanisms underlying disease activity.

In the present study, lymphocyte subsets, both T (CD3+, CD4+, and CD8+) and B (CD19+) cells were elevated in children with INS compared with healthy controls at time of relapse. These findings are consistent with the concept that immune activation plays an important role in the pathogenesis of nephrotic syndrome. Previous studies have reported abnormalities in T‐cell subsets in children with INS, suggesting that the dysregulation of T‐helper and cytotoxic T cells may contribute to the production of circulating permeability factors that affect podocyte structure and function [10, 11].

Among the lymphocyte subsets analyzed, cytotoxic T cells appeared to be particularly increased in both SSNS and SRNS groups. Our study showed significantly high counts of CD3+, CD4+, CD8+, and CD19+ cells in SRNS patients versus controls on the first visit, and only CD8+ cells were very high in SSNS patients. This is in line with other studies that reveal cytotoxic T cells possess a role in the generation of cytokines in SSNS and that CD8+ T cells play an integral part in immune‐mediated renal injury in nephrotic syndrome [12, 13]. Increased expression of IL‐13 mRNA in CD8+T cells has been observed during relapse of childhood nephrotic syndrome, indicating a potential role of these cells in disease activity. Experimental models have also demonstrated that overexpression of IL‐13 can induce podocyte injury and nephrotic‐like features, including albuminuria and hypoalbuminemia [14, 15]. While these findings support the involvement of cytotoxic T cells in nephrotic syndrome, their exact role in initiating the disease remains incompletely understood. Therefore, our results should be interpreted as suggesting an association between increased CD8+ T‐cell counts and INS rather than establishing a definitive causal relationship.

In addition to T‐cell alterations, our study also observed higher levels of CD19+ B lymphocytes in children with SRNS compared with controls at time of relapse. Role of B cells has gained increasing attention in INS, particularly in SRNS and frequently relapsing disease, and our results support this view. Increasing evidence indicates that B cells contribute to the pathogenesis of nephrotic syndrome not only through antibody production but also through cytokine secretion and interactions with T cells. B cells can also modulate immune responses by presenting antigens, producing proinflammatory cytokines, and influencing T‐cell activation and differentiation [16, 17]. Beyond antibody production, B cells exert important antibody‐independent functions via cytokine secretion and T‐cell costimulation [18]. A positive correlation of CD19+ counts with CD4+ and CD8+ counts observed in Pearson’s correlation analysis of our study strengthens this view. So, there is a possible role of CD8+ T cells in the initiation of the disease, and the progression and complications of INS are mediated by B cell and Th1 cells derived IFN‐γ and cytotoxic T cell or Th2 cell‐derived IL‐13 [19, 20].

Poststeroid increases in CD3+/CD19+ across groups, with selective CD8+ (SSNS) and CD4+ (SRNS) rises, suggest differential steroid impacts: immunosuppression in SSNS but resistance‐driven expansion in SRNS, as seen in prior T‐subset variances during steroid‐responsive states.

The analysis of urinary cytokines in our study revealed limited differences between groups at the time of diagnosis in contrary to previous study [10]. Our negative baseline findings may reflect timing of sampling, disease heterogeneity, or compartmentalization of cytokine production that is not fully captured in urine at a single time point. Interestingly, after steroid therapy, only SRNS patients showed a significant rise in urinary IFN‐γ and IL‐13, while SSNS children did not demonstrate major cytokine shifts, which is broadly compatible with a possible intrarenal inflammatory milieu in steroid‐resistant disease. IFN‐γ and IL‐13 are central mediators of Th1 and Th2 responses, respectively. Our observation that these cytokines increase despite systemic steroids in SRNS suggests probable ongoing local immune activation that is relatively steroid‐insensitive, complementing clinical evidence that prolonged or intensified steroids often fail to induce remission in this group.

ROC curve analysis was performed to evaluate the potential ability of lymphocyte subsets and urinary cytokines to differentiate disease groups. Though CD8+ T cells demonstrated moderate discriminatory ability, however, these results should not be interpreted as suggesting that such markers are necessary for diagnosing nephrotic syndrome in clinical practice [11].

The study had several limitations. First, the sample size was relatively small, which influences the subgroup analysis and overestimation of ROC curve diagnostic performance. We could not perform normalization of cytokine with respect to urinary creatinine, which is a limitation in interpreting results. We also could not perform the genetic testing in all children with SRNS. Finally, the study was conducted at a single center, which may limit the generalizability of the findings.

Despite these limitations, our study provides additional evidence that immune cell alterations are present in children with INS and may differ between steroid‐sensitive and steroid‐resistant diseases. These findings support the concept that complex interactions between T cells, B cells, and cytokines may contribute to disease pathophysiology. Further studies with larger cohorts, longitudinal follow‐up, and integrated immunological and genetic analyses are needed to better clarify the mechanisms underlying steroid responsiveness and to identify reliable biomarkers that may help guide individualized treatment strategies.

## Funding

This work was partially supported by research funds received as MD Thesis grant from the Indian Council of Medical Research (ICMR) with grant no. 3/2/Dec‐21/PG‐Thesis‐HRD [15] dated 18 April 2022.

## Disclosure

The authors have nothing to report.

## Ethics Statement

This study was performed in line with the principles of the Declaration of Helsinki. Approval was granted by the Institute Ethics Committee of AIIMS Bhubaneswar.

Ethical clearance has been obtained from the Institutional Ethics Committee (IEC) of AIIMS, Bhubaneswar with Approval Ref. no. IEC/AIIMS BBSR/PG Thesis/2021‐22/60 dated 13 August 2021.

## Conflicts of Interest

The authors declare no conflicts of interest.

## Data Availability

The data supporting this study are not publicly available due to privacy concerns regarding sensitive information about the children involved. Data are available from the corresponding author upon reasonable request.
